# Supplementary material for: Identification of photoperiod-induced specific miRNAs in the adrenal glands of Sunite sheep (Ovis aries)
Source: Front Vet Sci. 2022 Jul 22;9:888207. doi: 10.3389/fvets.2022.888207 (PMC9354845; doi:10.3389/fvets.2022.888207)
Supplement: Supplementary file 1 [file Data_Sheet_1.ZIP › Supplementary file/miRDeep2 file.zip!/miRDeep2 file/Know_Structure/SP42A1/oar-mir-194.pdf]

miRBase precursor : oar-mir-194
Total read count : 2440
oar-miR-194 read count : 2437
remaining reads : 3

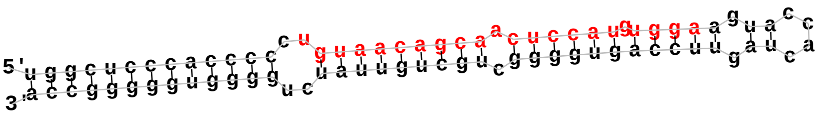

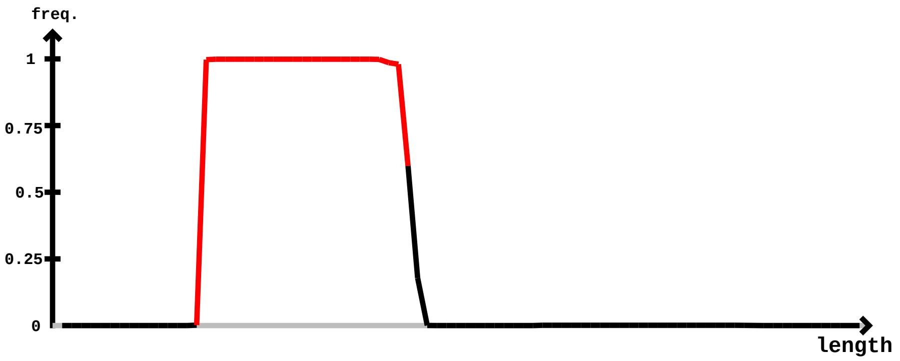


```
                 oar-miR-194
5'- uggcucccacccccuguaacagcaacuccaugugggaaguaccacuaguuccaguggggcugcuguuaucuggggugggggcca   -3'    exp
    ((((((((((((..(((((((((.((((((.(((((.((....)).))))))))))).)))))))))..))))))))))))    reads     mm        sample
    .............cuguaacagcaacuccaugugg................................................    3         0         seq
    .............cuguaacagcaacuccaugugga...............................................    1         0         seq
    ..............uguaacagcaacuccaug...................................................    2         0         seq
    ..............uguaacagcaacuccaugu..................................................    30        0         seq
    ..............uguaacagcaacuccaugug.................................................    14        0         seq
    ..............uguaacagcaacuccaugugg................................................    925       0         seq
    ..............uguaacagcaacuccaugugga...............................................    1023      0         seq
    ..............uguaacagcaacuccauguggaa..............................................    417       0         seq
    ..............uguaacagcaacuccauguggaC..............................................    17        1         seq
    ..............uguaacagcaacuccauguggaag.............................................    1         0         seq
    ...............guaacagcaacuccaugugg................................................    2         0         seq
    ...............guaacagcaacuccaugugga...............................................    2         0         seq
    .................................................ccaguggggcugcuguuaucu.............    1         0         seq
    .................................................ccaguggggcugcuguuaucug............    1         0         seq
    .................................................ccaguggggcugcuguuaucugg...........    1         0         seq
```
